# Current and Emerging Treatment Options for Hypertriglyceridemia: State-of-the-Art Review

**DOI:** 10.3390/ph18020147

**Published:** 2025-01-23

**Authors:** Jakub Michal Zimodro, Manfredi Rizzo, Ioanna Gouni-Berthold

**Affiliations:** 1 1st Chair and Department of Cardiology, Medical University of Warsaw, 02-097 Warsaw, Poland; 2 School of Medicine, Department of Health Promotion, Mother and Child Care, Internal Medicine and Medical Specialties (Promise), University of Palermo, 90133 Palermo, Italy; 3 Center for Endocrinology, Diabetes and Preventive Medicine, Faculty of Medicine, University Hospital Cologne, University of Cologne, Kerpener Str. 62, 50937 Cologne, Germany

**Keywords:** hypertriglyceridemia, apoC-III, ANGPTL3, small interfering ribonucleic acid, antisense oligonucleotide, FGF21

## Abstract

Hypertriglyceridemia (HTG) is associated with a residual risk of atherosclerotic cardiovascular disease. Extremely elevated triglyceride (TG) concentrations, particularly due to familial chylomicronemia syndrome (FCS), pose a risk for acute pancreatitis. Standard therapies with statins, fibrates, omega-3 fatty acids, and niacin may be insufficient to reduce elevated TG levels and improve clinical outcomes in patients with HTG. Novel antisense oligonucleotides and small interfering ribonucleic acids target the key modulators of TG-rich lipoprotein catabolism. Among apolipoprotein C-III (apoC-III) inhibitors, olezarsen and plozasiran appear to be safer alternatives for volanesorsen regarding the risk of drug-induced thrombocytopenia in patients with FCS or severe HTG. After the failure of vupanorsen, a new angiopoietin-like protein 3 (ANGPTL3) inhibitor, zodasiran, demonstrated the potential to decrease TG levels in patients with moderate HTG. Meanwhile, the fibroblast growth factor 21 (FGF21) analog, pegozafermin, became another candidate for the treatment of severe HTG. This comprehensive review outlines pharmacological targets in TG-rich lipoprotein metabolism, discusses international guidelines, and summarizes the latest evidence from clinical trials to provide insight into the current and emerging treatment options for primary HTG.

## 1. Introduction

Elevated plasma triglyceride (TG) concentration is associated with a residual risk of atherosclerotic cardiovascular disease (ASCVD) [1]. The prevalence of mild-to-moderate hypertriglyceridemia (HTG), defined as TG levels 150–499 mg/dL (1.7–5.6 mmol/L), is approximately 10% [2]. In contrast, the prevalence of severe HTG (sHTG), defined as TG levels >880 mg/dL (10 mmol/L), is estimated at 0.2%, while TG levels >1770 mg/dL [>20 mmol/L]) are observed in 0.014% of the population. sHTG poses a clinically significant risk for acute pancreatitis, accounting for approximately 10% of all cases [3]. As HTG-induced acute pancreatitis can lead to multiorgan failure and intensive care unit admission, in-hospital mortality can be up to 10% [4].

Mild-to-moderate HTG has a polygenic etiology with a strong influence of secondary factors, such as a fat-rich diet with a high glycemic index, excessive alcohol consumption, or metabolic syndrome [5]. Primary sHTG arises predominantly due to multifactorial chylomicronemia, resulting from large-effect heterozygous variants and accumulation of common small-effect polymorphisms [6]. Roughly 1–10 in a million patients develop monogenic sHTG with extremely elevated TG levels [7]. Familial chylomicronemia syndrome (FCS) is an autosomal recessive disorder characterized by absent lipolytic activity resulting from biallelic mutations in *LPL* (80% of cases), *APOC2*, *APOA5*, *LMF1,* and *GPIHBP1* [8]. Notably, acute pancreatitis occurs in approximately 80% of FCS patients [9], with 40% experiencing a mean of 13 episodes during their lifetime [10].

Among standard therapies, statins diminish cardiovascular risk yet only moderately reduce TG levels [11]. Conversely, fibrates and omega-3 fatty acids provide greater TG reductions, but their impact on ASCVD risk is not well established. Despite a TG-lowering effect, niacin does not improve cardiovascular outcomes, while lomitapide may be hepatotoxic. Overall, classical lipid-lowering therapies do not allow for robust TG reduction in patients with sHTG and are ineffective in those with FCS [12]. Therefore, innovative ribonucleic acid (RNA) interference-based therapies targeting lipolysis modulators, such as apolipoprotein C-III (apoC-III) and angiopoietin-like protein 3 (ANGPTL3), have been introduced [13]. Volanesorsen is already available in clinical practice in the European Union, United Kingdom, and Brazil, whereas olezarsen has been recently approved in the United States [14]. Plozasiran and zodasiran are still in development and demonstrated promising results in the recent phase 3 randomized controlled trials [14]. Meanwhile, fibroblast growth factor 21 (FGF21) analogs emerged as other potential candidates for TG-lowering drugs [15].

The aim of this work, based on a selective PubMed search for up-to-date evidence published by December 2024, is to provide insight into the current and emerging options for the treatment of primary HTG. This review (i) outlines triglyceride-rich lipoprotein (TRL) metabolism to elucidate molecular mechanisms targeted by TG-lowering therapeutics; (ii) discusses the American and European recommendations for HTG management, highlighting the efficacy and safety of traditional pharmacological treatments; and (iii) summarizes the latest data from clinical trials investigating apoC-III and ANGPTL3 inhibitors, as well as FGF21 analogs.

## 2. Pharmacological Targets in Triglyceride-Rich Lipoprotein Metabolism

**Figure 1:**
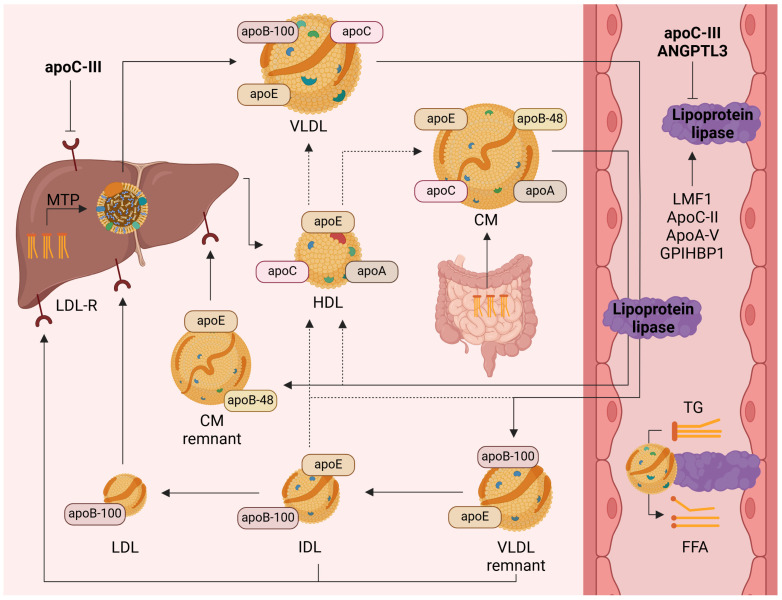
Metabolism of triglyceride-rich lipoproteins. ANGPTL3—angiopoietin-like 3; apo—apolipoprotein; CM—chylomicron; FFA—free fatty acid; GPIHBP1—glycosylphosphatidylinositol-anchored high-density lipoprotein binding protein 1; HDL—high-density lipoprotein; IDL—intermediate-density lipoprotein; LDL—low-density lipoprotein; LDL-R—low-density lipoprotein receptor; LMF1—lipase maturation factor 1; MTP—microsomal triglyceride transfer protein; TG—triglyceride, VLDL—very low-density lipoprotein. Created with BioRender.com.

As plasma TG are enclosed within lipoproteins, TG levels reflect the concentrations of all TRL [16]. The metabolism of TRL is demonstrated in Figure 1. Briefly, intestine-derived TG are transported in apoB-48-containing chylomicrons, while TG of hepatic origin are carried in very low-density lipoproteins (VLDL), which contain apoB-100 [16]. In the circulation, TG are hydrolyzed by lipoprotein lipase (LPL) anchored to the luminal surface of the vascular endothelium [17]. LPL secretion is facilitated by lipase maturation factor 1 (LMF1), while glycosylphosphatidylinositol-anchored high-density lipoprotein-binding protein 1 (GPIHBP1) reinforces LPL transcytosis [17]. Through LPL-mediated TG hydrolysis, adipose tissue and muscles are supplied with free fatty acids, which are either re-esterified and stored or directly oxidized as an energy source, respectively [18].

LPL activity is regulated by various proteins, with apoA-V and apoC-II acting as LPL activators, and apoC-III and ANGPTL3 as inhibitors [19]. While moderately elevated plasma TG result from the overproduction of VLDL and chylomicrons, severely elevated TG levels arise predominantly due to reduced LPL activity [20]. Both increased secretion and impaired hepatic clearance of TG induce the accumulation of cholesterol-enriched TRL remnants of <70 nm size, which can enter the subendothelial space and reinforce proatherogenic processes [20]. Furthermore, at extremely high TG levels, excessive amounts of free fatty acids generated by TG hydrolysis inhibit mitochondrial complexes in acinar cells, triggering cytokine release and tissue necrosis—a mechanism proposed to explain the pathogenesis of HTG-induced pancreatitis [21]. Due to the central role in TRL catabolism, LPL-mediated lipolysis has appeared as a novel treatment target for HTG [22].

## 3. Current Recommendations for the Management of Hypertriglyceridemia

Classification of HTG is inconsistent [23,24,25,26], as presented in Table 1. According to the American College of Cardiology and American Heart Association (ACC/AHA), an increase in ASCVD risk is driven by persistent primary HTG with non-fasting TG levels ≥ 175 mg/dL (≥2 mmol/L) in at least three serial measurements [23]. At the same time, the European Society of Cardiology and European Atherosclerosis Society (ESC/EAS) reported that fasting TG >150 mg (>1.7 mmol/L) are associated with an elevated ASCVD risk [24]. Consistently though, HTG diagnosis should be followed by ASCVD risk assessment and exclusion of secondary causes [23,24].

**Table 1:** Classification of normotriglyceridemia and hypertriglyceridemia [23,24,25,26].

Society	Classification	Triglyceride Levels [mg/dL (mmol/L)]
American College of Cardiology/American Heart Association (2018)	Normal	<175 (<2.0)
	Moderate	175–499 (2.0–5.6)
	Severe	≥500 (≥5.6)
European Society of Cardiology (2019)	Normal	<150 (<1.7)
	Mild-moderate	150–880 (1.7–10.0)
	Severe	≥880 (≥10.0)
European Atherosclerosis Society (2021)	Optimal	<100 (<1.2)
	Borderline	100–150 (1.2–1.7)
	Moderately elevated	150–500 (1.7–5.6)
	Severe	500–880 (5.6–10.0)
	Extreme	>880 (>10.0)
Endocrine Society (2012)	Normal	<150 (<1.7)
	Mild	150–199 (1.7–2.3)
	Moderate	200–999 (2.3–11.2)
	Severe	1000–1999 (11.2–22.4)
	Very severe	≥2000 (≥22.4)

As per ACC/AHA guidelines, TG levels ≥ 500 mg/dL (≥5.6 mmol/L) indicate the need for pancreatitis prevention [23]. In contrast, the ESC/EAS mentioned TG levels ≥ 880 mg/dL (≥10.0 mmol/L) but stressed that this higher threshold might potentially underestimate HTG as a pancreatitis risk factor [24]. Overall, lifestyle modification, consisting of dietary intervention, weight loss, and regular physical activity, remains the cornerstone of the treatment of primary HTG [25]. Additional TG reductions provided by standard lipid-lowering drugs are summarized in Table 2.

**Table 2:** Triglyceride-lowering effect of lipid-lowering drugs [5,27,28,29,30,31,32].

Drug	Expected Triglyceride Reduction
Statins	15–30%
Ezetimibe	5–10%
PCSK9 inhibitors	10–20%
Bempedoic acid	0–5%
Omega-3 fatty acids	20–50%
Fibrates	20–50%
Niacin	20–50%
Lomitapide	30–70%

### 3.1. Low-Density Lipoprotein-Lowering Drugs

According to the ACC/AHA, moderate HTG in patients at moderate ASCVD risk favors the initiation or intensification of statin treatment [23]. Furthermore, statins should be considered in patients with moderate ASCVD risk and sHTG [23]. Correspondingly, the ESC/EAS recommended statins in patients at high ASCVD risk with plasma TG > 200 mg/dL (>2.3 mmol/L) [24]. Foremost statins diminish global ASCVD risk due to reductions in low-density-lipoprotein cholesterol (LDL-C) levels and pleiotropic protective effects against endothelial dysfunction, plaque vulnerability, oxidative stress, and inflammation, among others [33]. Statin monotherapy decreases LDL-C levels by 25–55% in a dose-dependent manner [27]. In contrast, the impact on plasma TG is lower ranging from −15 to −30%, resulting from decreased VLDL production and accelerated VLDL clearance [27]. Therefore, only 20–50% of patients with elevated baseline TG ≥ 177 mg/dL (≥2 mmol/L) achieve on-statin TG < 150 mg/dL (<1.7 mmol/L), highlighting the need for additional TG-lowering therapy [27]. LDL-lowering agents other than statins are not specifically recommended in HTG patients, as their use is aimed at LDL-C levels and global ASCVD risk [24]. Of note, ezetimibe reduces TG levels by 5–10% [5], while a 10–20% decrease can be expected during the treatment with proprotein–convertase subtilisin/kexin type 9 inhibitors [28]. On the contrary, bempedoic acid has a neutral effect on plasma TG [29]. 

### 3.2. Omega-3 Fatty Acids

TG reductions ranging from approximately −20 to −30% can be obtained with omega-3 fatty acids, which suppress the incorporation of fatty acids into VLDL and enhance LPL activity [30]. In addition, omega-3 fatty acids are believed to exert an anti-atherosclerotic effect independently of TG reduction [30]. In the REDUCE-IT trial, purified eicosapentaenoic acid (EPA) lowered the risk of cardiovascular events by 25% among statin-treated, high-risk patients with elevated TG levels (135–500 mg/dL [1.5–5.6 mmol/L], median 216 mg/dL [2.4 mmol/L]) [34]. However, the trial faced criticism, as the results could have been affected by mineral oil used as a placebo [35]. Furthermore, the cardiovascular benefit was greater than expected with the median TG reduction of −18.3% [36]. According to the ESC/EAS, icosapent ethyl should be considered in high-risk patients with on-statin plasma TG 135–500 mg/dL (1.5–5.6 mmol/L) [24]. Conversely, the ACC/AHA stressed the need for omega-3 fatty acids intake only in patients with TG levels ≥ 500 mg/dL (≥5.6 mmol/L) [23], while a more recent AHA Scientific Statement concluded that EPA plus docosahexaenoic acid, or EPA-only at a daily dose of 4 g, is an effective and safe option for reducing TG levels either as monotherapy or an add-on to other TG-lowering agents [37].

### 3.3. Fibrates

Fibrates, peroxisome proliferator-activated receptor-alpha (PPARα) agonists, lower plasma TG by 20–50%, possibly through decreasing apoC-III production and increasing LPL activity [38]. According to the ESC/EAS, fenofibrate and bezafibrate may be considered in addition to statins in primary prevention settings and high-risk patients with TG levels > 200 mg/dL (>2.3 mmol/L) [24]. Nonetheless, the impact of fibrates on ASCVD risk is unclear. In the PROMINENT trial, pemafibrate, a new selective PPARα agonist, failed to reduce the risk of cardiovascular events among patients with type 2 diabetes and mild-to-moderate HTG (200–499 mg/dL [2.3–5.6 mmol/L], median 271 mg/dL [3.1 mmol/L]) [39]. In the ongoing ENSEMBLE trial, a triple combination of fenofibrate, ezetimibe, and moderate-intensity statin will be compared to statin-dose escalation in 3958 patients with TG levels 200–500 mg/dL (2.3–5.6 mmol/L), type 2 diabetes, and either ASCVD or risk factors for ASCVD [40]. The findings of previous studies with fenofibrate were inconclusive. In the FIELD study performed in patients with type 2 diabetes, fenofibrate did not improve the primary cardiovascular endpoint, coronary events, but decreased the total risk of cardiovascular events (−11%, *p* = 0.035), driven by the lower rates of non-fatal myocardial infarction (−24%, *p* = 0.01) and coronary revascularizations (−21%, *p* = 0.003) [41]. Furthermore, in the ACCORD lipid trial including 5518 patients with type 2 diabetes and median TG levels of 162 mg/dL (1.2 mmol/L), the combination of fenofibrate and simvastatin did not reduce the rates of fatal cardiovascular events, MI or stroke, compared to simvastatin alone [42]. A 5-year follow-up confirmed the original neutral effect of fenofibrate in the overall ACCORD lipid cohort [43]. Correspondingly, in the BIP trial, bezafibrate failed to reduce the composite of primary endpoint of fatal or non-fatal MI and sudden death in 3090 patients with a previous MI or stable angina and mean TG levels of 145 mg/dL (1.6 mmol/L) [44]. However, in a post-hoc analysis, the risk of the primary endpoint significantly decreased by 39.5% in bezafibrate-treated patients with baseline TG ≥ 200 mg/dL (>2.3 mmol/L) [44], while a sub-study revealed a positive correlation between risk reduction and the extent of TG reduction [45]. Moreover, a 20-year mortality follow-up of the BIP participants showed that the risk significantly decreased by 25% in subjects with HTG (≥200 mg/dL [≥2.3 mmol/L]) but not in those with TG levels < 200 mg/dL (>2.3 mmol/L) [46]. Overall, a recent meta-analysis of 12 randomized controlled trials reported an association between fibrate treatment and a lower risk of cardiovascular events, yet the risk reduction was attributable to LDL-C rather than the TG-lowering effect [47].

### 3.4. Niacin

Extended-release niacin can reduce plasma TG by 20–50%, but its clinical efficacy and safety are questionable [31]. The AIM-HIGH trial with extended-release niacin [48] and the HPS2-THRIVE trial with niacin plus laropiprant [49], both performed in patients with ASCVD, demonstrated no cardiovascular benefits and an increased frequency of serious adverse events. No medication containing niacin is currently approved in Europe [24].

### 3.5. Lomitapide

The ESC/EAS suggested that lomitapide may be considered in cases of sHTG [24]. An inhibitor of microsomal triglyceride transfer protein (MTP) suppresses the production and secretion of TRL and is approved in patients with homozygous familial hypercholesterolemia (HoFH) [50]. In a study performed in 18 FCS patients, lomitapide reduced plasma TG by 70%, with 13 patients achieving TG levels ≤ 750 mg/dL (≤8.5 mmol/L) and none experiencing on-treatment pancreatitis [32]. Concurrently, four patients had transaminases elevated >3 times the upper limit of the normal range, and nine had increased hepatic fat [32]. Moreover, two cases reporting lomitapide use in FCS patients described a progression of hepatic steatosis, raising safety concerns [51,52]. Hence, no randomized controlled trials with lomitapide have been performed in patients with sHTG.

## 4. New Approaches to Triglyceride-Lowering Therapy

### 4.1. Apolipoprotein C-III Inhibitors

ApoC-III is an abundant component of TRL serving as an LPL inhibitor [53]. It is speculated that apoC-III either directly binds to LPL or restricts its access to TG [53]. In addition, apoC-III disrupts apoB-100 and apoE-mediated binding to LDL receptors, thereby suppressing hepatic clearance of TRL remnants [54]. Loss-of-function mutations in *APOC3* are associated with reduced TG levels and ASCVD risk [55]. Therefore, apoC-III inhibition has emerged as a possible treatment for HTG.

#### 4.1.1. Volanesorsen

**Figure 2:**
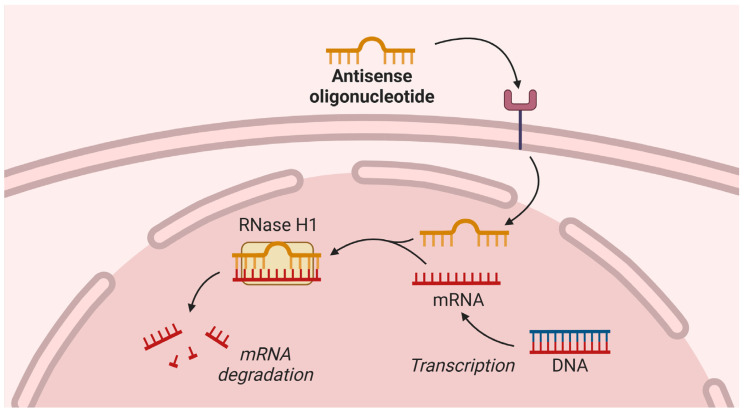
Mechanism of action of antisense oligonucleotides. DNA—deoxyribonucleic acid; mRNA—messenger ribonucleic acid; RNase—ribonuclease. Created with BioRender.com.

Volanesorsen is a second-generation 2′-O-methoxyl-modified antisense oligonucleotide (ASO) [56]. In hepatocytes, volanesorsen binds to *APOC3* messenger RNA (mRNA) and induces its degradation by ribonuclease H1 [56]. The mechanism of action of ASO is presented in Figure 2. Consequently, volanesorsen lowers plasma apoC-III by up to 80% in a prolonged and dose-dependent manner [57].

The phase 3 COMPASS trial was performed in 114 individuals with plasma TG ≥ 500 mg/dL (≥5.6 mmol/L) [58]. At 3 months, the mean TG levels of 1261 mg/dL (14.2 mmol/L) decreased by 71.2% (*p* < 0.0001) in those who received 300 mg of volanesorsen weekly. Notably, 49% of trial participants had polygenic scores for TG at the ≥95th percentile and 19% carried loss-of-function mutations in *LPL*, *APOC2,* or *APOA5*, while 6% had genetically confirmed FCS. Volanesorsen lowered plasma TG by 73, 69, and 73% in all subsets, respectively. Simultaneous reductions occurred in chylomicron TG, apoB-48, and VLDL-C, whereas HDL-C and apoA-I increased (*p* < 0.0001 for all). LDL-C was elevated by 95.5%, yet the overall number of atherogenic particles did not change, as non-HDL-C decreased and apoB remained unaltered <116 mg/dL. Regarding safety, five events of acute pancreatitis occurred during the treatment period, all in the placebo group. Mild-to-moderate injection-site reactions were the most common adverse event, which occurred in 24% of volanesorsen injections.

The phase 3 APPROACH trial included 66 patients with genetically confirmed FCS [59]. At 3 months, TG levels decreased by 77% (*p* < 0.001) in the volanesorsen group. The mean absolute TG reduction reached −1712 mg/dL (−19.3 mmol/L). In line with the results of the COMPASS trial, volanesorsen reduced chylomicron TG, apoB-48, VLVD-C, non-HDL-C, and apoC-III but increased LDL-C, HDL-C, and apoA-I (*p* < 0.001 for all) independently of genotype or baseline HTG treatment. The incidence of acute pancreatitis was not a prespecified endpoint. Thrombocytopenia was commonly reported, yet with a biweekly platelet count assessment and thresholds of <100,000/μL for dose reduction and <50,000/μL for dose interruption, no declines to <50,000/μL occurred. Overall, thrombocytopenia might arise following volanesorsen administration but normalizes after treatment cessation.

The long-term efficacy and safety of volanesorsen were assessed in the APPROACH Open-Label Extension (OLE) trial [60]. TG reductions at 3, 6, 12, and 24 months reached −48, −55, −50, and −50%, respectively, in 44 participants of the APPROACH trial, −65, −43, −42, and −66% in 5 participants of the COMPASS trial, and −60, −51, −47, and −46% in 19 volanesorsen-naïve subjects. The TG-lowering effect became less robust with time, presumably due to thrombocytopenia-driven dose reductions and interruptions necessary in 65% and 74% of participants, respectively. Severe adverse events were reported in 34% of patients, with thrombocytopenia being the most common cause of drug discontinuation. On-treatment platelet count decreased in 23.5% of patients, while 61.8% experienced injection-site reactions. Markedly, 82 events of acute pancreatitis occurred in 33 participants within 5 years prior to the treatment initiation, whereas only 5 events occurred in 5 patients during the treatment period. Moreover, among 12 volanesorsen-naïve patients treated in the Early Access to Medicines Scheme in the United Kingdom, 47–55% TG reductions were maintained through 15 months, while 10 pre-treated patients experienced 10–38% decreases throughout the 21 months of follow-up [61]. Lower long-term reductions were likely the result of a transition from weekly to biweekly dosages. The rates of acute pancreatitis decreased by 74%, with one event per 2.8 years within 5 years prior to the treatment initiation, and one event per 11 years during the treatment period.

Correspondingly, the BROADEN trial included 40 subjects with familial partial lipodystrophy, hepatic steatosis, glycated hemoglobin > 7%, and plasma TG ≥ 500 mg/dL (≥5.6 mmol/L) [62]. In volanesorsen-treated patients, 3-month TG reductions reached −88%, while the 12-month reduction in the hepatic fat fraction was as high as −53%. Of note, individual analysis of the COMPASS, APPROACH, and BROADEN trials revealed that volanesorsen exerts a favorable effect on hepatic fat and might potentially prevent steatohepatitis in patients with HTG of different etiology [63].

Finally, a recent fixed-effect meta-analysis of three randomized trials (APPROACH, COMPASS, and BROADEN) with 207 subjects confirmed an association between volanesorsen and a reduced risk of acute pancreatitis [64]. A history of acute pancreatitis was reported in 40 and 51% of patients in the volanesorsen and placebo groups, respectively. In contrast, the rates of on-treatment pancreatitis were 2 and 10%, respectively. Notably, on-treatment pancreatitis in the volanesorsen group occurred only within 4 months from the first dose but throughout the entire treatment period in the placebo group.

#### 4.1.2. Olezarsen

Olezarsen is a next-generation ASO targeting *APOC3* mRNA [65]. A conjugation to N-acetylgalactosamine facilitates its selective uptake by the asialoglycoprotein receptor 1 in the liver [65]. In the latest phase 1 trial performed in 28 healthy Japanese Americans with plasma TG ≥ 90 mg/dL (≥1 mmol/L), a maximum TG reduction was achieved with 60 mg single-dose olezarsen reaching −52.7% at 15 days and −73.8% at 92 days [66].

A phase 2 trial randomized 114 high-risk patients with elevated fasting TG (200–500 mg/dL [2.3–5.6 mmol/L] and median 262 mg/dL [2.96 mmol/L]) [67]. At 6 months, olezarsen, in doses of 10 mg weekly, 15 mg biweekly, and 10 or 50 mg every 4 weeks, led to significant TG reductions of −23 (*p* < 0.004), −56, −60, and −60% (*p* < 0.0001), respectively. Notably, 91% of patients treated with 50 mg of olezarsen every 4 weeks achieved plasma TG < 150 mg/dL (<1.7 mmol/L), and 46% of all olezarsen-treated patients achieved values <100 mg/dL (<1.1 mmol/L). Moreover, olezarsen significantly reduced VLDL-C, non-HDL-C, apoB, and apoC-III, and increased HDL-C and apoA-I. Mean changes in LDL-C levels varied from −1 to 19%, while the latter was associated with alterations or interruptions in LDL-lowering therapy. Total TRL levels decreased by 51%, with the highest reductions in the concentrations of large- and medium-size particles (−68 and −63%, respectively, *p* < 0.0001) [68]. In addition, large-size LDL levels increased (186%, *p* = 0.0034), while the opposite was observed for small-size LDL particles (−39%, *p* = 0.0713), suggesting that olezarsen favorably affects the atherogenic profile of circulating lipoproteins [68]. The most common adverse event, injection-site erythema, affected 15.6% of patients in the treatment group, but there were no cases of platelet count <100,000/μL and no hepatic or renal abnormalities [67].

The phase 2b Bridge-TIMI 73a trial was performed in 154 individuals with either moderate HTG (150–499 mg/dL) and elevated ASCVD risk or sHTG (≥500 mg/dL [≥5.6 mmol/L]) [69]. At 6 months, olezarsen in a monthly dose of 50 and 80 mg reduced median TG levels of 241.5 mg/dL (2.7 mmol/L) by 57.1 and 60.9%, respectively. Hence, 86 and 93% of patients with moderate HTG reached TG levels < 150 mg in the 50 mg and 80 mg groups, respectively. Both treatment regimens led to significant reductions in VLDL-C, non-HDL-C, remnant cholesterol, apoB, and apoC-III, while LDL-C levels remained unchanged. Serious adverse events occurred in only 8% of participants, with no relation to the study treatment. Elevated transaminases were more common in the olezarsen group as compared to the placebo. However, the difference was driven by elevations of <3 times the upper limit of the normal range. Changes in platelet count and glycated hemoglobin were comparable between the treatment and placebo groups.

The phase 3 Balance trial included 66 patients with genetically confirmed FCS [70]. Compared to the placebo, mean TG levels of 2630 mg/dL (29.7 mmol/L) significantly decreased by 43.5 percentage points in the 80 mg olezarsen group at 6 months (*p* < 0.001), but not in the 50 mg group. Since the co-primary endpoint was nonsignificant, all secondary endpoints were formally nonsignificant as well. However, apoC-III was numerically lower in the 80 mg group compared to placebo at 6 and 12 months (−73.7 and −81.3 percentage points, respectively), as was non-HDL-C (−17.7%) and apoB-48 (−84%). In the 80 mg group, mean baseline LDL-C levels increased from 22.8 to 37.6 mg/dL, while apoB, from 58.4 to 69.0 mg/dL, remaining low, as consistently reported in FCS patients. Eleven episodes of acute pancreatitis had occurred by week 53 in the placebo group, and one episode had occurred in each olezarsen group (rate ratio for pooled olezarsen groups vs. placebo, 0.12; 95% confidence interval, 0.02–0.66). Severe adverse events affected 18% of patients in the 80 mg group, but there were no meaningful hepatic, renal, or platelet abnormalities.

#### 4.1.3. Plozasiran

**Figure 3:**
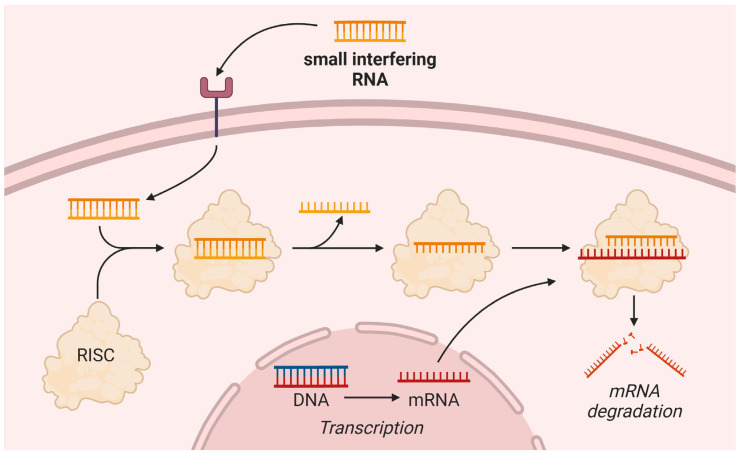
Mechanism of action of small interfering ribonucleic acids. DNA—deoxyribonucleic acid; mRNA—messenger ribonucleic acid; RISC—ribonucleic acid-induced silencing complex; RNA—ribonucleic acid. Created with BioRender.com.

Plozasiran (ARO-APOC3) is an N-acetylgalactosamine-conjugated small interfering RNA (siRNA) [71]. While volanesorsen and olezarsen interfere with mRNA in the nucleus, plozasiran potently inhibits cytoplasmatic *APOC3* mRNA [71]. The mechanism of action of siRNA is presented in Figure 3. A phase 1 trial was performed in 52 healthy volunteers, 40 patients with HTG (TG levels ≥ 300 mg/dL [≥3.4 mmol/L]), and 20 patients with chylomicronemia (TG levels ≥ 800 mg/dL [9.9 mmol/L]) [72]. At 113 days, plozasiran reduced apoC-III by 62.0–94.4% and TG levels by 65.6–81.0%. Ten of 88 plozasiran-treated patients experienced mild-to-moderate transaminase elevations, which returned to baseline by the end of the trial, while no platelet decreases occurred.

The phase 2 SHASTA-2 trial studied 226 subjects with TG levels 500–4000 mg/dL (5.6–45 mmol/L) [73]. At 24 weeks, mean TG levels of 897 mg/dL (10.1 mmol/L) decreased by 49, 53, and 57% in the 10, 25, and 50 mg plozasiran groups, respectively (*p* < 0.001 for all). Markedly, 90.6% of the plozasiran-treated patients reached TG levels < 500 mg/dL (<5.6 mmol/L) within 24 weeks, and 76.5% maintained these levels through 48 weeks. Simultaneously, plozasiran reduced non-HDL-C, remnant cholesterol, and apoB-48 and increased HDL-C and apoA-I. LDL-C levels increased by 60% in those receiving the highest dose (50 mg) but steadily decreased after 24 weeks and were not significantly different from the placebo at week 48. Adverse events were comparable between the treatment and placebo groups and were associated with comorbidities rather than the study treatment. Glycemic control worsened only in patients with preexisting diabetes, while acute pancreatitis occurred only in those with a history of acute pancreatitis (two [3.3%] cases in the placebo group vs. one [0.6%] case in the treatment group).

The phase 2b MUIR trial included 353 individuals, with mixed hyperlipidemia defined as TG levels 150–499 mg/dL (1.7–5.6 mmol/L), and either LDL-C levels ≥ 70 mg/dL or non-HDL-C ≥ 100 mg/dL [74]. Compared to the placebo at 24 weeks, mean TG levels of 244 mg/dL (2.75 mmol/L) were reduced by −49.8% with 10 mg of plozasiran quarterly, −56.0 with 25 mg quarterly, −62.4 with 50 mg quarterly, and −44.2 with 50 mg half-yearly (*p* < 0.001 for all). TG reductions were strongly associated with declines in apoC-III and remnant cholesterol levels. In addition, all doses of plozasiran lowered apoB and LDL-C levels. Platelet count decreases and transaminase elevations were not observed, while glycemic control worsened in approximately 20% of patients in the 50 mg-quarterly and 50 mg-half-yearly groups. At high doses of plozasiran, increased substrate delivery to the liver led to increased hepatic gluconeogenesis, potentially explaining glycated hemoglobin elevations.

The phase 3 PALISADE trial was performed in 75 patients with FCS or symptomatic persistent chylomicronemia with plasma TG > 1000 mg/dL (>11.3 mmol/L) [75]. At 10 months, reductions in median TG levels of 2044 mg/dL (23.0 mmol/L) reached −80 and −78% in the 25 and 50 mg plozasiran groups, respectively (*p* < 0.001). Simultaneously, baseline non-HDL-C decreased, while HDL-C and LDL-C increased. However, LDL-C remained within the range of <55 mg/dL in all groups. The response to plozasiran was independent of genetic status or sex. Notably, plozasiran-treated patients had lower odds of acute pancreatitis (odds ratio, 0.17; 95% confidence interval, 0.03–0.94, *p* = 0.03). Adverse events were comparable between the treatment and placebo groups. At 12 months, mean glycated hemoglobin was similar to the baseline levels yet increased in patients with preexisting diabetes or prediabetes. Alanine aminotransferase transiently increased in 23 and 46% of patients in the 25 and 50 mg groups, respectively, but remained ≤ 3 times the upper limit of the normal range.

### 4.2. Angiopoietin-like 3 Inhibitors

ANGPTL3 belongs to a family of glycoproteins secreted from hepatocytes and acts as an LPL inhibitor [76]. One of the potential inhibitory mechanisms involves postprandial binding to ANGPTL8 with subsequent enhancement of LPL cleavage [77]. In fact, ANGPTL3/8 complex acts as a 100-fold-more-potent inhibitor of LPL activity compared to ANGPTL3 alone [78]. Individuals with loss-of-function mutations in *ANGPTL3* have lower TG, LDL-C, HDL-C, and non-HDL-C levels, accompanied by a 40% reduction in ASCVD risk, as compared to noncarriers [79]. Therefore, ANGPTL3 has been investigated as a new target for both HTG and hypercholesterolemia treatment.

#### 4.2.1. Zodasiran

Zodasiran (ARO-ANG3) is an N-acetylgalactosamine-conjugated siRNA that targets *ANGPTL3* mRNA [80]. A phase 1 basket trial was performed in 52 healthy individuals with fasting TG > 100 mg/dL (>1.1 mmol/L) and LDL-C levels > 70 mg/dL. At 85 days, dose-dependent TG reductions ranging from −16.6 to −54.4% occurred in the single-dose zodasiran groups. Correspondingly, TG decreased by maximally 72.0% in the repeated-dose groups at 113 days. Simultaneous declines in VLDL-C, non-HDL-C, and apoB levels were reported in both groups. LDL-C reductions in the repeated-dose groups ranged from −34.4 to −44.5% with 300 and 200 mg of zodasiran, respectively. In contrast, changes in LDL-C levels in the single-dose groups varied between −26.9 and 4.1% with 100 and 200 mg of zodasiran, respectively. HDL-C levels decreased by up to 12.9% in the single-dose groups and by up to 37.2% in the repeated-dose groups, possibly resulting from enhanced HDL clearance, as ANTPTL3 inhibition leads to de-inhibition of endothelial lipase. There were no serious adverse events related to the study treatment, including thrombocytopenia and hepatotoxicity.

The phase 2 ARCHES-2 trial included 204 patients with mixed hyperlipidemia defined as fasting TG 150–499 mg/dL (1.7–5.6 mmol/L) and either LDL-C ≥ 70 mg/dL or non-HDL-C ≥ 100 mg/dL [81]. Compared to the placebo at 24 weeks, dose-dependent reductions in mean TG levels of 246 mg/dL [2.8 mmol/L] reached −51, −57, and −63 percentage points with 50, 100, and 200 mg of zodasiran, respectively, administered on day 1 and week 12. As a result, 88% of patients receiving 200 mg of zodasiran achieved fasting TG levels < 150 mg/dL (1.7 mmol/L). Parallel declines in ANGPTL 3 levels positively correlated with changes in LDL-C and non-HDL-C levels. Of note, the LDL-lowering effect was attenuated in participants at high tertiles for baseline TG levels, likely due to rapid TG hydrolysis. In addition, plozasiran reduced HDL-C, remnant cholesterol, lipoprotein (a), and apoB at all doses. Similar rates of adverse events occurred in the treatment and placebo groups, with no platelet count decreases or transaminase elevations. There was a transient elevation in HbA1c levels in patients with preexisting diabetes who received the highest dose of zodasiran (200 mg), but insulin sensitivity did not change. Interestingly, the incidence of urinary tract infections in the 200 mg group reached 12% compared to 4% in the placebo group, yet all events occurred in patients with a history of diabetes.

#### 4.2.2. Evinacumab

Evinacumab is a human monoclonal antibody directed against circulating ANGPTL3 [82]. Primarily, evinacumab reduces LDL-C levels by up to 50% [83]. Its long-term efficacy and safety have been proven in patients with HoFH, for which it is an approved treatment [84]. In phase 1 trials performed on 83 subjects with mixed dyslipidemia, evinacumab lowered TG levels by approximately 70% [85]. In contrast to patients with mild-to-moderate HTG, TG reductions in patients with plasma TG >1500 (>17 mmol/L) ranged from −0.9 to −93.2%, indicating an impact of genetic status [85].

In a phase 2 trial including 51 individuals with sHTG, evinacumab did not lower TG levels among patients with genetically confirmed FCS over 12 weeks [86]. Conversely, significant TG reductions occurred in patients with heterozygous loss-of-function LPL pathway mutations (−64.8 vs. 9.4%, *p* = 0.0076) or multifactorial chylomicronemia without LPL pathway mutations (−81.7 vs. 80.9%, *p* = 0.0418), as compared to the placebo. Therefore, LPL activity seems compulsory for the ANGPTL3 inhibitor to reduce TG levels. Simultaneously, remnant cholesterol and apoA-I decreased in all cohorts, with a trend towards higher LDL-C and lower apoC-III, apoB-100, and apoB-48 levels. Evinacumab was well tolerated, as no substantial differences in adverse events occurred between the treatment and placebo groups. There were no elevations in transaminases or hepatic fat. Twenty-five events of acute pancreatitis occurred during the study treatment, which was attributable to inadequate TG control. Nevertheless, a phase 2 trial aiming to investigate evinacumab in acute pancreatitis prevention was terminated due to poor recruitment [11].

#### 4.2.3. Vupanorsen

Vupanorsen is a second-generation, N-acetylgalactosamine-conjugated ASO targeting the *ANGPTL3* mRNA [87]. A phase 2 trial was performed in 105 patients with fasting TG > 150 mg/dL (>1.7 mmol/L), type 2 diabetes, and hepatic steatosis [87]. At 6 months, vupanorsen reduced TG levels by up to 53%, with 80 mg administered subcutaneously every 4 weeks (*p* < 0.001). In addition, ANGPTL3, apoC-III, remnant cholesterol, total cholesterol, non-HDL-C, and apoB decreased, while LDL-C levels did not change. However, as compared to placebo, patients treated with 80 mg of vupanorsen experienced significant elevations in transaminases and hepatic fat fraction. Correspondingly, vupanorsen reduced TG levels by 60% in four patients with familial partial lipodystrophy [88]. In this study, compared to the baseline, all participants experienced a 19.7% increase in hepatic fat fraction at 13 weeks, followed by a −1.1% decrease at 27 weeks. The former was accompanied by a transient transaminase elevation of <3 times the upper limit of the normal range. In a phase 2b trial performed in 286 statin-treated patients with plasma TG 150–500 mg/dL (1.7–5.6 mmol/L) and non-HDL-C ≥ 100 mg/dL, vupanorsen reduced TG levels by up to −56.8% [89]. Elevations in alanine and aspartate aminotransferases were as high as 33.3 and 44.4%, respectively. Furthermore, there was a dose-dependent increase in the hepatic fat fraction of up to 76%. Therefore, the clinical development of vupanorsen has been discontinued due to hepatotoxicity [11].

#### 4.2.4. Monoclonal Antibody Against Angiopoietin-like-3/8 Complex

LY3475766, a monoclonal antibody against ANGPTL3/8 complex, was investigated in a phase 1 trial including 68 individuals with TG levels > 135 mg/dL (1.5 mmol/L) and LDL-C levels ≥ 70 mg/dL [90]. Over 28 days, dose-dependent TG, LDL-C, and apoB reductions reached −70, −35, and −31%, respectively, while HDL-C increased by up to 25%. No serious adverse events occurred during the study. However, larger randomized controlled trials have not been performed. Similarly, an antibody directed against ANGPTL8 has been tested in preclinical models only [91].

### 4.3. Fibroblast Growth Factor 21 Analogs

**Figure 4:**
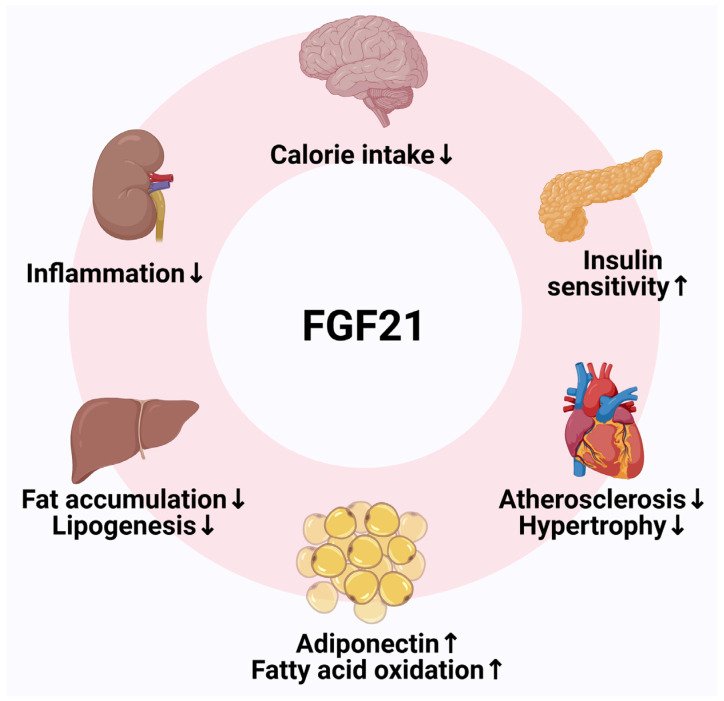
Metabolic effects of fibroblast growth factor 21 (FGF21). Created with BioRender.com.

FGF21 is a key regulator of glucose and lipid metabolism, with a beneficial effect on insulin sensitivity and body weight [92,93]. Metabolic effects of FGF21 are summarized in Figure 4. FGF21 promotes the expression of genes involved in fatty acid oxidation, as well as MTP and apoB, thus accelerating VLDL production [94]. At the same time, FGF21 facilitates VLDL-derived free fatty acid uptake into adipose tissue [94]. In addition, FGF21 stimulates LPL-mediated TRL disposal in adipose tissue and accelerates hepatic clearance of VLDL remnants, leading to lower non-HDL-C levels [94]. Consequently, FGF21 reduced the area and severity and improved the stability of atherosclerotic lesions in a mice model [95]. FGF21 analogs, such as pegozafermin, were developed with glycoPEGylation technology, which prolongs the half-life and limits off-target FGF21 effects [96].

Among 64 participants with body mass index 30–45 kg/m^2^ and plasma TG 150–500 mg/dL (1.7–5.6 mmol/L), pegozafermin lowered TG levels by 54% and increased HDL-C levels by 36% over 12 weeks [97]. Notably, adiponectin increased by 103%, possibly contributing to TG reduction. The phase 2 ENTRIGUE trial randomized 67 patients with elevated plasma TG (500–2000 mg/dL [5.6–22.6 mmol/L], median 622 mg/dL [7 mmol/L]) [98]. At 8 weeks, median TG reductions in the pegozafermin groups ranged from −36.4 to −63.4%. Thereby, 79.7% of the pegozafermin-treated patients reached TG levels < 500 mg/dL (<5.6 mmol/L). The TG-lowering effect was independent of baseline treatment and diabetic status. Concurrent declines occurred in non-HDL-C, apoB, and apoC-III levels. As compared to the placebo, pegozafermin administered in a weekly dose of 27 mg subcutaneously was associated with 44.5% higher HDL-C levels and a 73% decrease in apoB48 levels, indicating improved clearance of chylomicrons and their remnants. Pegozafermin was well tolerated, with no serious adverse events and mild-to-moderate gastrointestinal disturbances being the most common adverse event. The 52-week efficacy and safety of pegozafermin will be assessed in the ongoing phase 3 ENTRUST trial, enrolling 360 patients with sHTG (500–2000 mg/dL [5.6–22.6 mmol/L]) [99].

## 5. Conclusions and Future Directions

Mild-to-moderate HTG is associated with an increased ASCVD risk [100], while sHTG is associated with an increased risk of acute pancreatitis [3]. Current guideline-recommended treatments [23,24] are limited and seem insufficient to achieve adequate TG reductions in patients with HTG, particularly sHTG [11]. Lifestyle measures, including a low-fat diet, require long-term compliance but rarely lead to normal TG levels [11]. The management of ASCVD risk in patients with mild-to-moderate HTG is based on statins that exert only a moderate TG-lowering effect [23,24,27]. Due to the limitations of the REDUCE-IT trial [34], there are no large, randomized controlled trials clearly showing that any TG-lowering drug can decrease the risk of either ASCVD or acute pancreatitis. Novel RNA interference-based therapies allow for robust TG reduction, as summarized in Table 3.

Volanesorsen demonstrated long-term effectiveness in patients with sHTG irrespectively of genetic status, but the European Medicines Agency (EMA) approved it only for the treatment of FCS [11]. Volanesorsen is also approved in the United Kingdom and in Brazil but not in the United States since the Food and Drug Administration (FDA) found that the potential side effects, including drug-induced thrombocytopenia, outweigh its benefits [11]. In contrast, olezarsen provides satisfactory TG reductions and decreases the incidence of acute pancreatitis with a good safety profile [67,68,69,70]. Thus, it is most likely going to replace volanesorsen. In December 2024, the FDA approved olezarsen as an adjunct to diet to reduce TG levels in adults with FCS [101], whereas the EMA issued an orphan designation for FCS treatment in August 2024 [102]. In addition, as olezarsen lowers the concentrations of atherogenic lipoproteins [68], it might also be beneficial in patients with moderate HTG to decrease the residual ASCVD risk. Plozasiran is another apoC-III inhibitor that showed the potential to reduce TG levels in patients with mixed hyperlipidemia and the risk of acute pancreatitis in patients with sHTG [73,74,75]. Similarly to olezarsen, plozasiran seems a safer alternative for volanesorsen. Hence, it received orphan designation for FCS treatment from the EMA, alongside a breakthrough therapy designation granted by the FDA in September 2024 [103]. However, the potential for increasing HbA1c levels [73,74,75], a signal not observed with olezarsen, requires further investigation. A phase 3 cardiovascular endpoint trial with plozasiran has been announced [104].

Although ANGPTL3 inhibition seemed a reasonable strategy to treat HTG, evinacumab failed to reduce TG levels in patients with FCS who lack LPL activity [85,86]. Based on these findings, zodasiran has been investigated in subjects with mild-to-moderate HTG [80,81]. In contrast to abandoned vupanorsen, zodasiran did not trigger serious adverse events and even reduced hepatic fat. Nonetheless, ANGPTL3 inhibitors can lower the concentrations of atherogenic particles independently of LDL receptor function and, hence, may be used in mixed hyperlipidemia and HoFH [13]. Conversely, as apoC-III inhibitors reduce TG levels irrespectively of LPL activity, they are effective in both moderate and severe HTG, including FCS. However, apoC-III inhibition does not affect or even increases LDL-C and apoB, which may diminish cardiovascular benefits and pose the need for parallel LDL-lowering therapy. Nonetheless, the further development of zodasiran has been put on hold by its developer, Arrowhead Pharmaceuticals, so they can focus on the marketing of plozasiran. The ongoing randomized controlled trials are presented in Table 4.

FGF21 analogs, such as pegozafermin, may be another drug class to lower TG levels but also to exert a hepatoprotective and antidiabetic effect, which requires further investigation [97,98,99]. Although alipogene tiparvovec has been withdrawn from the market, genetic approaches to the treatment of LPL deficiency are tested in preclinical models and might expand the armamentarium for FCS in the future [107]. Due to the rapid development in this field, effective HTG therapy may be on the horizon. Large, randomized cardiovascular outcome trials are needed to confirm whether the TG-lowering effects of apoC-III and ANGPTL3 inhibitors translate into clinical benefits.

## Figures and Tables

**Table 3:** Ribonucleic acid interference-based triglyceride-lowering therapies. Data presented as the highest least-square mean percent change in triglyceride levels from baseline or least-square mean difference in change vs. placebo (% or percentage points) with the corresponding variations in the lipid profiles. ANGPTL3—angiopoietin-like 3; Apo—apolipoprotein; ASCVD—atherosclerotic cardiovascular disease; FCS—familial chylomicronemia syndrome; HDL-C—high-density lipoprotein cholesterol; LDL-C—low-density lipoprotein cholesterol; LPL—lipoprotein lipase; TG—triglyceride.

Trial	Population	TG Reduction	Lipid Profile Changes
**Volanesorsen**			
COMPASS Phase 3 [58]	Multifactorial chylomicronemia or FCS with TG ≥ 500 mg/dL (≥5.6 mmol/L) N = 114	−71.2% at 3 months with 300 mg	ApoC-III: −76.1% Non-HDL-C: −27.3% ApoB: +5.8% LDL-C: +95.5% HDL-C: +61.2%
APPROACH Phase 3 [59]	FCS N = 67	−76.5% at 3 months with 300 mg	ApoC-III: −84.2% Non-HDL-C: −45.9% ApoB: +19.5% LDL-C: +135.6% HDL-C: +46.1%
**Olezarsen**			
Phase 2 [67]	TG 200–500 mg/dL (2.3–5.6 mmol/L) with established ASCVD or high ASCVD risk N = 114	−62% at 6 months with 50 mg	ApoC-III: −74% Non-HDL-C: −20% ApoB: −10% LDL-C: +10% HDL-C: +30%
Bridge-TIMI 73a Phase 2b [69]	TG 150–499 mg/dL (1.7–5.6 mmol/) with increased ASCVD risk, or TG ≥ 500 mg/dL N = 154	−53.1 at 6 months with 80 mg	ApoC-III: −73.2 Non-HDL-C: −23.1 ApoB: −18.5 LDL-C: −7.7 HDL-C: +39.6
Balance Phase 3 [70]	FCS N = 66	−43.5 at 6 months with 80 mg	ApoC-III: −73.7 ApoB-48: −84.0 Non-HDL-C: −24.2
**Plozasiran**			
SHASTA-2 Phase 2 [73]	TG 500–4000 mg/dL (5.6–45 mmol/L) N = 226	−57.0% at 24 weeks with 50 mg	ApoC-III: −77.4% Non-HDL-C: −20.2% ApoB: −7.3% LDL-C: +60.3% HDL-C: +57.0%
MUIR Phase 2b [74]	TG 150–499 mg/dL (1.7–5.6 mmol/) with LDL-C ≥ 70 mg/dL or non-HDL-C ≥ 100 mg/dL N = 353	−62.4 at 24 weeks with 50 mg quarterly	ApoC-III: −78.5 Non-HDL-C: −24.2 ApoB: −19.1 LDL-C: −13.5 HDL-C: +45.8
PALISADE Phase 3 [75]	TG >1000 mg/dL (>11.3 mmol/L) with FCS, low LPL activity or a history of acute pancreatitis N = 75	−59.0 at 10 months with 25 mg	ApoC-III: −91
**Zodasiran**			
Phase 1 [80]	TG > 100 mg/dL (>1.1 mmol/L) and LDL-C > 70 mg/dL N = 52	−58.6% at 85 days with 300 mg	ANGPTL3: −84.3% Non-HDL-C: −17.6% ApoB: −9.4% LDL-C: −9.1% HDL-C: −18.6%
Phase 2 ARCHES-2 [81]	TG 150–499 mg/dL (1.7–5.6 mmol/L) with LDL-C ≥ 70 mg/dL or non-HDL-C ≥ 100 mg/dL N = 204	−63.1 at 24 weeks with 200 mg	ANGPTL3: −73.3 Non-HDL-C: −36.4 ApoB: −21.9 LDL-C: −19.9 HDL-C: −24.5

**Table 4:** Current randomized controlled trials with ribonucleic acid interference-based triglyceride-lowering therapies [105,106]. HTG—hypertriglyceridemia; sHTG—severe hypertriglyceridemia; TG—triglyceride.

Trial	Condition	Estimated End Date
	**Olezarsen**	
Phase 3 NCT05185843	FCS	January 2025
ESSENCE CS9—TIMI 73b Phase 3 NCT05610280	TG 200–500 mg/dL (2.3–5.6 mmol/L) with established ASCVD or high ASCVD risk, or TG ≥ 500 mg/dL (≥5.6 mmol/L)	March 2025
CORE—TIMI 72a Phase 3 NCT05079919	sHTG TG ≥ 500 mg/dL (≥5.6 mmol/L)	April 2025
CORE2 CS6—TIMI 72b Phase 3 NCT05552326	sHTG TG ≥ 500 mg/dL (≥5.6 mmol/L)	June 2025
CORE-OLE Phase 3 NCT05681351	sHTG TG ≥ 500 mg/dL (≥5.6 mmol/L)	May 2026
Phase 3 NCT05185842	FCS previously treated with volanesorsen	June 2027
	**Plozasiran**	
MUIR-3 Phase 3 NCT06347133	HTG TG 150–499 mg/dL (1.7–5.6 mmol/L)	October 2026
SHASTA-3 Phase 3 NCT06347003	sHTG TG ≥ 500 mg/dL (≥5.6 mmol/L)	October 2026
SHASTA-4 Phase 3 NCT06347016	sHTG TG ≥ 500 mg/dL (≥5.6 mmol/L)	October 2026

## Data Availability

Not applicable.
